# Association of vitamin C, vitamin D, vitamin E and risk of bladder cancer: a dose-response meta-analysis

**DOI:** 10.1038/srep09599

**Published:** 2015-04-23

**Authors:** Fuqiang Chen, Qingshu Li, Yang Yu, Wenrong Yang, Fei Shi, Yan Qu

**Affiliations:** 1 Department of Pain Management, Qingdao Municipal Hospital, Qingdao, China; 2 Intensive Care Unit, Qingdao Municipal Hospital, Qingdao, China

## Abstract

A dose-response meta-analysis was conducted to assess the association of vitamin C, D, E with risk of bladder cancer. Pertinent studies were identified in PubMed and Embase. The random-effect model was used. The relative risk (95% confidence interval) of bladder cancer was 0.99 (0.95–1.03) for every 100 IU/day increment in vitamin D from diet plus supplement and 0.95 (0.90–1.00) for every 10 nmol/L increment in circulating vitamin D. The effect for every 10 mg/day increment was 0.96 (0.90–1.02) for vitamin E from diet plus supplement, 0.83 (0.72–0.95) from diet and 0.88 (0.67–1.15) from supplement, and the effect was 0.84 (0.76–0.94) for every 1 mg/dL increment in circulating α-Tocopherol and 1.22 (1.00–1.49) for every 0.1 mg/dL increment in circulating γ-Tocopherol. The observed association for vitamin D and vitamin E was significant among smokers but not among non-smokers. No significant association was found between vitamin C and risk of bladder cancer in the dose-response analysis. Based on the dose-response analysis, the risk of bladder cancer might be inversely associated with vitamin D and E (especially α-Tocopherol), but positively associated with γ-Tocopherol.

Worldwide, 386,300 new cases and 150,200 deaths from bladder cancer were estimated to occur in 2008, and the majority of bladder cancer occurs in males^1^. The American Cancer Society presents 4 recommendations to reduce cancer risk, including maintain a healthy weight, adopt a physically active lifestyle, consume a healthy diet with an emphasis on plant foods, and limit alcoholic consumption^2^. Smoking and occupational exposures are the major risk factors of bladder cancer in Western countries, whereas chronic infection with Schistosoma hematobium in developing countries^1^. Vitamin D receptors have been detected in superficial transitional cell carcinoma of the human bladder^3^, and vitamin D inhibits proliferation and induces apoptosis in human bladder tumor cells in vitro^4^. Vitamin C and vitamin E are also supposed to have a protective effect against bladder development through their actions as an antioxidant and free radical scavenger^5^. Many epidemiology studies have been conducted to assess the association of Vitamin C^6^,^7^,^8^,^9^,^10^,^11^,^12^,^13^,^14^,^15^,^16^,^17^,^18^,^19^,^20^, D^7^,^16^,^21^,^22^,^23^,^24^,^25^ and E^7^,^8^,^9^,^10^,^12^,^13^,^14^,^15^,^16^,^17^,^18^,^19^,^26^,^27^,^28^,^29^ with risk of bladder risk, but the results are not consistent. Therefore, we performed a dose-response meta-analysis to quantitatively summarize the evidence from epidemiological studies on the association of Vitamin C, vitamin D and vitamin E with risk of bladder cancer.

## Results

### Literature search and study characteristics

The flow chart for study inclusion is shown in supplementary Figure 1. For vitamin C from diet plus supplement, 8 studies in 7 articles^7^,^8^,^10^,^15^,^16^,^17^,^20^ (2 studies in 1 article^20^ by sex) were included involving 2,021 cases among 194,443 participants. For vitamin C from diet, 14 studies in 12 articles^6^,^8^,^9^,^11^,^12^,^14^,^15^,^17^,^18^,^19^,^20^,^30^ (2 studies in 2 articles^20^,^30^ by sex) were included involving 5,765 cases among 292,002 participants. For vitamin C from supplement, 9 studies from 8 articles^8^,^10^,^13^,^14^,^16^,^17^,^18^,^20^ (2 studies in 1 article^20^ by sex) were included involving 3,331 cases among 1,199,984 participants. For circulating vitamin C, only 1 article^6^ was identified.

For vitamin D from diet plus supplement, 3 articles^7^,^16^,^25^ were included involving 842 cases among 49,156 participants. For circulating vitamin D, 4 articles^21^,^22^,^23^,^24^ were included involving 1,737 cases among 12,944 participants. No articles were identified for vitamin D from diet only or supplement only.

For vitamin E from diet plus supplement, 6 articles^7^,^8^,^10^,^15^,^16^,^17^ were included involving 1,760 cases among 194,182 participants. For vitamin E from diet, 9 studies from 8 articles^8^,^9^,^12^,^14^,^15^,^17^,^19^,^30^ were included involving 2,985 cases among 275,265 participants. For vitamin E form supplement, 7 articles^8^,^10^,^13^,^14^,^16^,^17^,^18^ were included involving 3,070 cases among 1,199,723 participants. For circulating α-Tocopherol, 4 articles^26^,^27^,^28^,^29^ were included involving 614 cases among 1,256 participants. For circulating γ-Tocopherol, 3 articles^26^,^27^,^28^ were included involving 579 cases among 1,151 participants. The detailed characteristics of the included studies are shown win supplementary table 1.

### Quantitative Synthesis

#### Vitamin D and bladder cancer (Table 1 and Figure 2–3)

[Fig f2]Although no association was found between bladder cancer and vitamin D from diet plus supplement, an inverse association was found or indicated with circulating vitamin D overall [0.75 (0.57–0.99), I2 = 51.7%], in cohort studies [0.82 (0.61–1.11), I2 = 46.3%] and case-control studies [0.55 (0.36–0.85), n = 1]. No publication bias was found for vitamin D from diet plus supplement (P = 0.41) and for circulating vitamin D (P = 0.87), and no individual study had an excessive influence in sensitivity analysis, respectively. Because only 3 articles for vitamin D from diet plus supplement and 4 articles for circulating vitamin D were included, subgroup analysis was not conducted further.

**Figure 3:**
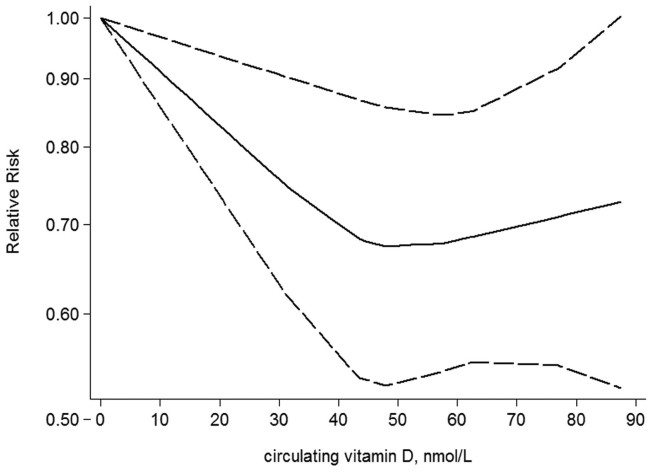
Dose-response analysis for circulating vitamin D and risk of bladder cancer.

The risk of bladder cancer was 0.99 (0.95–1.03), P_for nonlinearity_ = 0.78 for every 100 IU/day increment of vitamin D from diet and supplement, and 0.95 (0.90–1.00), P_for nonlinearity_ = 0.10 for every 10 nmol/L increment of circulating vitamin D (Figure 3) (Table 1).

## Discussion

To our knowledge, this is the first dose-response meta-analysis to quantitatively summarize the evidence on vitamin C, vitamin D and vitamin E and risk of bladder cancer. In this meta-analysis, vitamin D and vitamin E (especially α-Tocopherol) were found inversely associated with risk of bladder in a linear dose-response manner, and the association was stronger among smokers. Vitamin C might not be associated with risk of bladder cancer. However, γ-Tocopherol might be positively associated with risk of bladder cancer.

Categories of the three micronutrients (vitamin C, vitamin D, and vitamin E) levels differed between the included studies, which might complicate the interpretation of the pooled results across study populations with different categories (i.e. pooling the results comparing highest vs. lowest categories^31^,^32^). In this respect, a dose–response meta-analysis with restricted cubic spline functions provides a solution to this problem from which the dose-response relationship (linear or non-linear) was first assessed and then a summary risk estimate can be derived for a standardized increase of the micronutrients levels^33^. We also assessed the associations between circulating vitamin C levels, circulating α-Tocopherol levels, circulating γ-Tocopherol, diet and supplement vitamin D intakes and risk of bladder cancer, which are not assessed in the available meta-analysis^31^,^32^. In addition, this meta-analysis found that the observed association for vitamin D and vitamin E was significant among smokers but not among non-smokers, which was also not assessed in the available meta-analysis^31^,^32^.

Several plausible mechanisms have been proposed by which vitamin E and vitamin C may delay various steps in carcinogenesis through its actions as an antioxidant and free radical scavenger^5^,^34^,^35^, including limiting free radical induced DNA damage and the formation of carcinogens such as N-nitroso compounds, decreasing the concentration of the bladder carcinogen 3-hydroxanthranilic acid, inhibiting the carcinogenic effect of saccharin and cancer cell growth, and stimulating immune function and apoptosis. Vitamin D is not an antioxidant, but its activity in preventing cancer development involves regulation of adherence and signaling, inhibition of proliferation, differentiation, cell cycle stabilization, promotion of apoptosis, and anti-neoangiogenesis^36^. In this meta-analysis, the observed association was more prominent in smokers. This finding is in line with the hypothesis that smokers may benefit more from vitamin D and vitamin E intake than nonsmokers, because the high antioxidant content and anticancer effects of vitamins may reduce the oxidative damage caused by cigarette smoking^37^, and current smokers have significantly lower concentrations of serum vitamin E than nonsmokers^38^. There was a 32% reduction in prostate cancer incidence and a 41% reduction in prostate cancer mortality after 6.1 years of treatment of α-Tocopherol in the Alpha-Tocopherol, Beta Carotene Cancer Prevention Trial including 29,133 male smokers^39^, although this benefit was attenuated during posttrial follow-up^40^. However, the benefit on prostate cancer with vitamin E supplementation was not observed in the Physicians' Health Study II Trial^41^,^42^ and the Selenium and Vitamin E Cancer Prevention Trial^43^,^44^, and both trials involved participants of very low levels of smoking. These findings on prostate cancer could also help understand the finding that vitamin E selectively protected against bladder cancer in smokers in this meta-analysis. In this meta-analysis, γ-Tocopherol might increase the risk of bladder. Although the mechanism has yet to be determined, the residual confounding by smoking cannot be ruled out, because smoking decreases some plasma antioxidants but increases γ-tocopherol levels^45^,^46^.

Although no association was found for vitamin C from diet plus supplement and vitamin C from supplement, an inverse association was found for vitamin C from diet. However, the inverse association was mainly caused by the results from case-control studies, and no association was indicated in cohort studies [1.02 (0.85–1.23)], and this was also the case for vitamin E. The distinction between the results from case-control and cohort studies might be attributed to the differences in study design considering prospective cohort studies do not suffer from recall bias and are anticipated to be less likely to have selection bias relative to case-control studies and are also believed to provide better evidence for causality in which vitamins intake precedes bladder incidence compared with case-control studies. For vitamin D, although no association was found with vitamin D from diet plus supplement, an inverse association was found with circulating vitamin D. This discrepancy might arise from the fact that self-reported dietary intake of vitamin D is a less accurate measure of vitamin D status than are circulating concentrations, because vitamin D is produced endogenously in response to sun exposure. Among the 4 articles included for circulating vitamin D, an inverse association was found or indicated in 3 articles^21^,^23^,^24^, but an increase (although not significant) risk of bladder associated with higher circulating vitamin D levels was found in 1 article^22^. As explained in the article^22^, this difference may be explained by the inclusion of women and nonsmokers in the current analysis, as a modest inverse association was also found when restricting the analysis to male smokers^22^. In addition, although the departure from a non-linearity was not significant (P_for nonlinearity_ = 0.78) that might be caused by the relatively small number of studies, the shape of the dose-response analysis suggested that the increased risk of bladder cancer was most pronounced at levels of circulating vitamin D less than 50 nmol/L. Importantly, the distribution of circulating vitamin D levels was higher in the PLCO study^22^, and few participants (about 43%) had circulating vitamin D levels < 50 nmol/L than those in the other studies (73%^24^ and 74%^23^). Therefore, the different distributions of circulating vitamin D levels across studies may make comparison between studies difficult and this could also contribute to the between-study heterogeneity. For vitamin E, although the observed association was mainly evident in case-control studies, circulating α-Tocopherol was also found inversely associated with risk of bladder cancer, and no heterogeneity (I2 = 0.00%) was found across studies.

Other factors that might influence the observed association should be considered. The inverse association between circulating vitamin D and risk of bladder cancer was stronger among men with lower vitamin D binding protein (DBP) [low DBP: 0.47 (0.23–1.00), high DBP: 0.83 (0.40–1.75)]^23^, and among low-FGFR3 expressers^24^. In addition, season of blood collection, physical activity, α-Tocopherol supplementation and time from blood collection to case diagnosis were also found to influence the association^22^,^24^. No association was found between circulating vitamin C and bladder cancer by prognostic subgroups (aggressive and nonaggressive)^6^. The association between circulating α-Tocopherol and bladder cancer risk differed by vitamin E supplement [users: 0.48 (0.28–0.83), non-users: 1.76 (0.71–4.35)]; however, the effect of total vitamin E on bladder cancer risk was 0.49 (0.22–1.07) among non-users and 0.84 (0.44–1.59) among users of α-Tocopherol and beta carotene supplement in another study^12^. The limited data precluded a more robust assessment in this meta-analysis.

Other limitations should also be of concern. First, although we extracted the RRs that reflected the greatest degree of control for potential confounders, the extent to which they were adjusted and the possibility that the observed association was due to unmeasured or residual confounding should be considered. Therefore, the observed association for α-Tocopherol, γ-tocopherol, and vitamin D should be confirmed by randomized controlled trials. One randomized controlled trial^42^ (the results were updated^41^) was identified, and no significant association was found between vitamin E supplement [bladder cancer death: 0.79 (0.33–1.88), bladder cancer: 1.21 (0.76–1.94)] and vitamin C supplement [bladder cancer death: 0.92 (0.39–2.17), bladder cancer: 0.85 (0.53–1.36)]. Second, misclassification and inaccurate measurement of vitamins intake should be of concern in observational studies. Third, although no publication bias was found, or the results did not change with the trim and fill method, validity of publication bias test should be questioned because of small number of studies included, especially for vitamin D, α-Tocopherol and γ-tocopherol.

In conclusion, vitamin D and vitamin E (especially α-Tocopherol) might be inversely associated with bladder cancer risk, while γ-tocopherol might be positively associated with bladder cancer risk. These results need to be confirmed in randomized controlled trials.

## Methods

### Literature search and selection

We performed a literature search up to September 2014 using the databases of Pubmed and Embase, using the following search terms *(bladder cancer) AND (((((((((Ascorbic Acid) OR vitamin C) OR Vitamin E) OR Tocopherol) OR vitamin D) OR 1,25-dihydroxyvitamin D) OR 25-hydroxyvitamin D) OR 25-hydroxyvitamin D3) OR 25-hydroxyvitamin D2)* without restrictions. We also reviewed the reference lists from retrieved articles to search for further relevant studies.

For inclusion, studies must fulfill the following criteria: (1) exposure of interest was Vitamin C, vitamin D or vitamin E; (2) outcome of interest was bladder cancer; (3) relative risk (RR) or odds ratio with 95% confidence interval (CI) was provided (we presented all results with RR for simplicity), or data available to calculate them; (4) conducted in humans; (5) for dose–response analysis, the number of cases and participants or person-years for each category of vitamins must also be provided (or data available to calculate them). The most recent study was included for duplicate publications.

### Data extraction

The following data were extracted: first author, study design, cohort name and follow-up duration for cohort studies, publication year, country where the study was conducted, participants' mean age, number of cases and total participants, methods for the measurement of vitamins intake, RR (95% CI) and adjusted covariates. For dose–response analysis, the number of cases and participants (or person-years) and RR (95% CI) for each category of vitamins were also extracted. We assigned the median level of vitamins for each category to each corresponding RR estimate. If the highest category of the studies was open-ended, we assumed that the boundary had the same amplitude as the adjacent category.

### Statistical Analysis

We first pooled the study-specific logarithms of RR for the highest versus lowest level of vitamins levels, using a random-effects model. Between studies heterogeneity was evaluated using I2 statistic, and I2 values of 25%, 50%, and 75% represent low, moderate, and high heterogeneity^47^, respectively. To explore sources of heterogeneity among studies, we conducted meta-regression analysis. A sensitivity analysis was conducted by omitting one study at each turn and recalculating the pooled estimates for the remainder of the studies to evaluate whether the results could have been affected markedly by a single study. Publication bias was assessed by Egger test, and the trim and fill method^48^ was used to incorporate theoretical missing studies when publication bias was detected.

We then conducted a two stage random effects dose response meta-analysis^49^. First, a restricted cubic spline model was estimated with a generalised least squares regression with 3 knots at percentiles 25%, 50%, and 75% of the distribution of vitamins levels, then the estimated study-specific 2 regression coefficients (3 knots minus 1) were combined in a multivariate random-effects meta-analysis. A test for a non-linear relation was calculated by making the coefficient of the second spline equal to zero. We used STATA version 12.0 (StataCorp LP, College Station, TX) to analyse the data, and P < 0.05 was considered statistically significant.

## Supplementary Material

Supplementary Information Supplementary Information

## Figures and Tables

**Figure 1:**
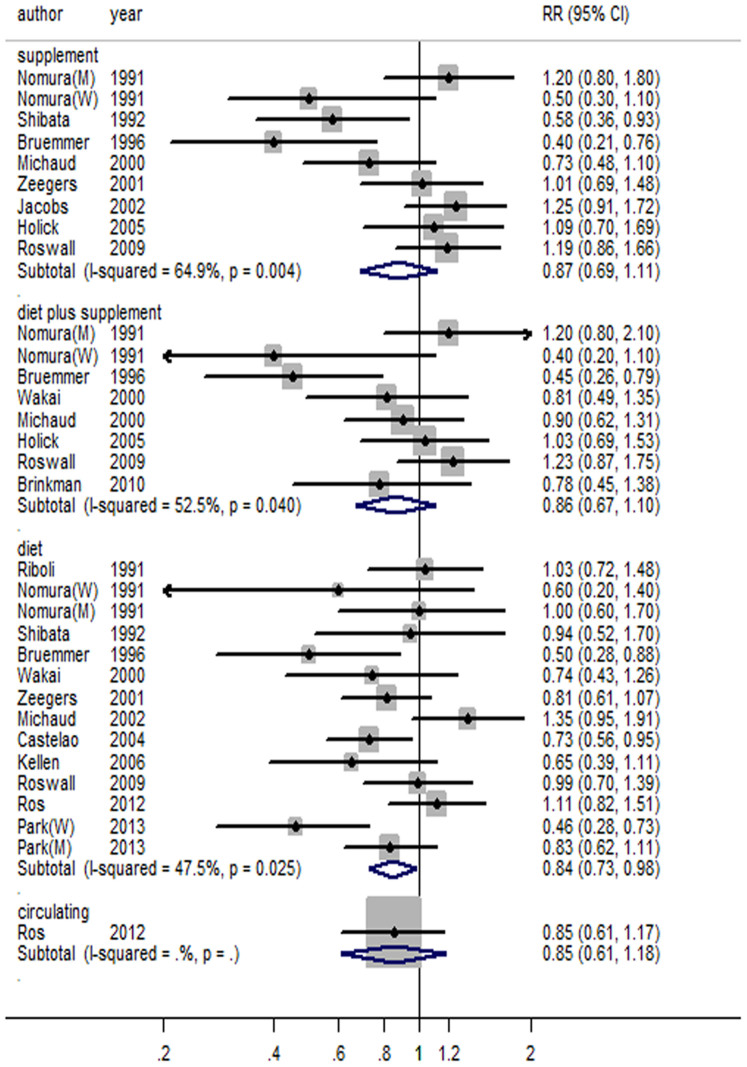
Forest plot for vitamin C and risk of bladder cancer.

**Figure 2 f2:**
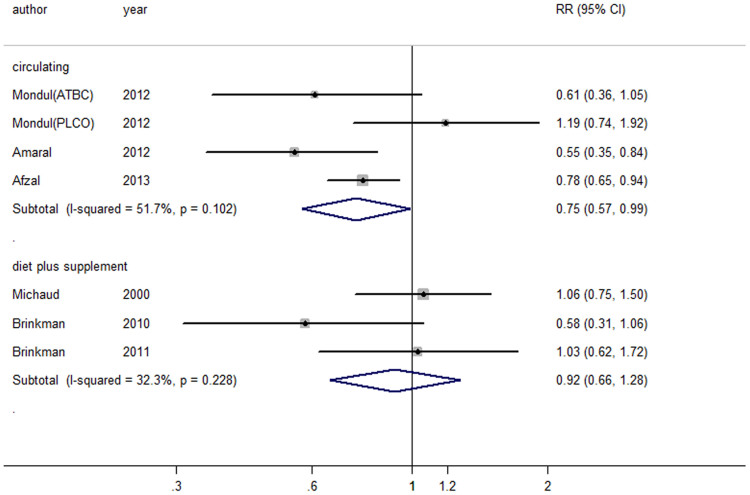
Forest plot for vitamin D and risk of bladder cancer.

**Figure 4:**
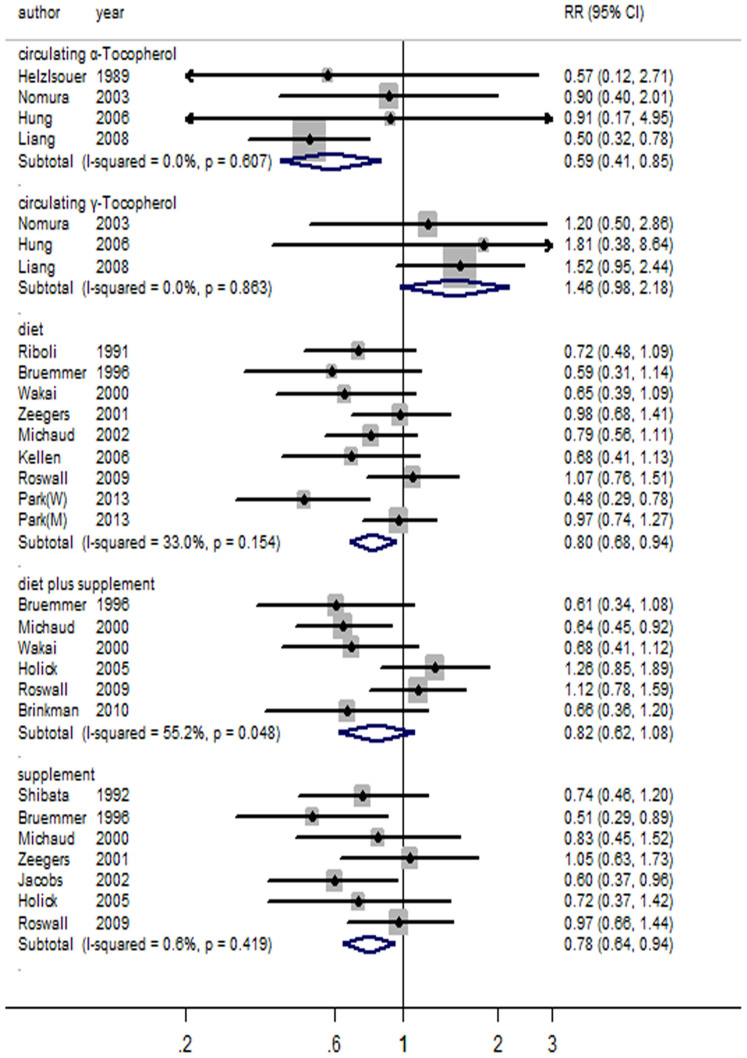
Forest plot for vitamin E and risk of bladder cancer.

**Figure 5:**
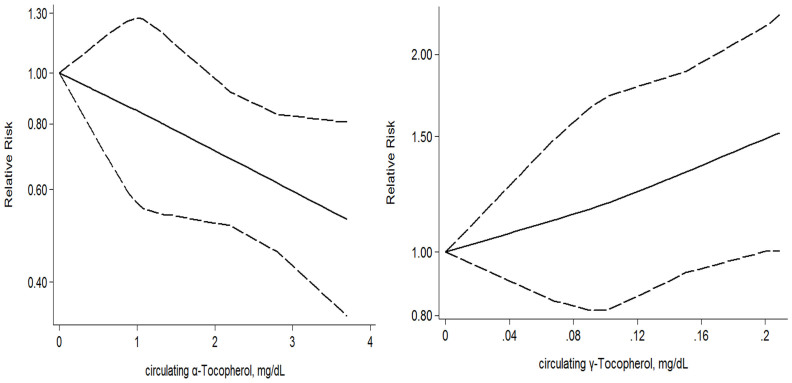
Dose-response analysis for circulating α-Tocopherol and circulating γ-Tocopherol risk of bladder cancer.

**Table 1:** Dose-response analysis on vitamin C, vitamin D and vitamin E with risk of bladder cancer

	N^a^	N (cases)	Dose increment	RR (95% CI)	P_for nonlinearity_
Vitamin C (diet plus supplement)	7	1617	every 100 mg/day	0.96 (0.90–1.02)	0.95
Vitamin C (diet)	8	3757	every 100 mg/day	0.93 (0.82–1.05)	0.13
Vitamin C (supplement)	4	843	every 100 mg/day	0.94 (0.85–1.03)	0.51
Vitamin C (circulating)	1	856	every 10 umol/L	0.96 (0.91–1.02)	0.76
Vitamin D (diet plus supplement)	3	840	every 100 IU/day	0.99 (0.95–1.03)	0.78
Vitamin D (circulating)	3	1739	every 10 nmol/L	**0.95 (0.90–1.00)**	0.10
Vitamin E (diet plus supplement)	5	1401	every 10 mg/day	0.96 (0.90–1.02)	0.73
Vitamin E (diet)	4	1057	every 10 mg/day	**0.83 (0.72–0.95)**	0.31
Vitamin E (supplement)	2	582	every 10 mg/day	0.88 (0.67–1.15)	0.26
α-Tocopherol (circulating)	4	614	every 1 mg/dl	**0.84 (0.76–0.94)**	0.97
γ-Tocopherol (circulating)	3	579	every 0.1 mg/dL	**1.22 (1.00–1.49)**	0.85

a: number of studies.
