# Supplementary material for: Association of vitamin C, vitamin D, vitamin E and risk of bladder cancer: a dose-response meta-analysis
Source: Sci Rep. 2015 Apr 23;5:9599. doi: 10.1038/srep09599 (PMC5386108; doi:10.1038/srep09599)
Supplement: Supplementary Information [file srep09599-s1.pdf]

Title: Association of vitamin C, vitamin D, vitamin E and risk of bladder cancer: a dose-response meta-analysis

Authors: Fuqiang Chen, Qingshu Li, Yang Yu, Wenrong Yang, Fei Shi, Yan Qu

Journal: Scientific Reports

Supplementary table 1 characteristics of the studies included in this meta-analysis

Author Country	Design, Study name	Age (mean, years)	Subjects (cases), sex	Follow -up (years)	Exposure (assessment method)	RR (95% CI) for the highest vs. lowest categories	Covariates adjusted
Helzlsouer et al. 1989, USA[1]	Cohort (NCC) Washington County project	58.9	105 (35), both	12	---	a-Tocopherol (circulating) 0.57 (0.12-2.71)	Sex, race, within 2 h for the interval between blood collection and the previous meal, cigarette smoking and vitamin supplements
Nomura et al. 1991, USA[2]	Matched case-control (PCC)	66.0 (men) 70.4 (women)	522 (261), both	---	Interview	Vitamin C (diet)<br>Men: 1.0 (0.6-1.7)<br>Women: 0.6 (0.2-1.4)<br>Vitamin C (supplement)<br>Men: 1.2 (0.8-1.8)<br>Women: 0.5 (0.3-1.1)<br>Vitamin C (total)<br>Men: 1.2 (0.8-2.1)<br>Women: 0.4 (0.2-1.1)	Sex, age, ethnic group, and pack-years of cigarette smoking.
Riboli et al. 1991, Spain[3]	Matched case-control (HCC)	---	1221 (432), men	---	Interview	Vitamin C (diet)<br>1.03 (0.72-1.48)<br>Vitamin E (diet)<br>0.72 (0.48-1.09)	Age, area of residence
Shibata et al. 1992, USA[4]	Cohort	74.9	11580 (71), men	70159 person-years	FFQ	Men:<br>Vitamin C (diet)<br>0.94 (0.52-1.70)<br>Vitamin C (supplement)<br>0.58 (0.36-0.93)<br>Vitamin E (supplement)	Age and smoking

						0.74 (0.46-1.20)	
Bruemmer et al. 1996, USA[5]	Case-control (PCC)	58.3 (cases) 57.1 (controls)	667 (262), both	---	Interview (supplement) FFQ (diet)	Vitamin C (diet) 0.50 (0.28-0.88) Vitamin C (supplement) 0.40 (0.21-0.76) Vitamin C (total) 0.45 (0.26-0.79) Vitamin E (diet) 0.59 (0.31-1.14) Vitamin E (supplement) 0.51 (0.29-0.89) Vitamin E (total) 0.61 (0.34-1.08)	Age, sex, county, smoking, and calories (for diet and total)
Michaud et al. 2000, USA[6]	Cohort, HPFS	40-75	47909 (320), men	12	FFQ	Vitamin C (total) 0.90 (0.62-1.31) Vitamin E (total) 0.64 (0.45-0.92) Vitamin D (total) 1.06 (0.75-1.50) Vitamin C (supplement) 0.73 (0.48-1.10) Vitamin E (supplement) 0.83 (0.45-1.52)	Age, energy, pack-years of smoking history, current smoking status, geographic region of the United States, total fluid intake, and cruciferous vegetable intake, and total fluid intake (for supplement)
Wakai et al. 2000, Japan[7]	Matched case-control (HCC)	66.6 (cases) 66.5 (controls)	592 (297), both	---	Interview	Vitamin C (diet) 0.74 (0.43-1.26) Vitamin E (diet) 0.65 (0.39-1.09) Vitamin C (total) 0.81 (0.49-1.35)	Sex, age, hospital, smoking and occupational history as a cook

						Vitamin E (total) 0.68 (0.41-1.12)	
Zeegers et al. 2001, Netherlands [8]	Cohort, Netherlands Cohort Study	62.7 (cases) 61.4 (controls)	3692 (569), both	6.3	FFQ	Vitamin C (diet) 0.81 (0.61-1.07)<br>Vitamin E (diet) 0.98 (0.68-1.41)<br>Vitamin C (supplement) 1.01 (0.69-1.48)<br>Vitamin E (supplement) 1.05 (0.63-1.73)	Age, sex, cigarette smoking amount and cigarette smoking duration
Jacobs et al. 2002, USA [9]	Cohort, Cancer Prevention Study II Cohort	Main (40-70)	991522 (1289), both	18	FFQ	Vitamin C (supplement) 1.25 (0.91-1.72)<br>Vitamin E (supplement) 0.60 (0.37-0.96)	Age, sex (in combined-sex models), educational level, cigarette smoking (current or former status and number of pack-years), consumption of citrus fruits/juices, consumption of vegetables, and vitamin C and vitamin E supplement use
Michaud et al. 2002, Finland [10]	Cohort, ATBC cohort	50-69	27111 (344), men smokers	11	FFQ	Vitamin C (diet) 1.35 (0.95-1.91)<br>Vitamin E (diet) 0.79 (0.56-1.11)	Age, duration of smoking, smoking dose, total energy, and trial intervention (alpha-tocopherol and beta-carotene supplements)
Nomura et al. 2003, USA [11]	Cohort (NCC), none	73.2	219 (109), men	>20	---	α-Tocopherol (circulating): 0.90 (0.40-2.01)<br>γ-Tocopherol (circulating): 1.20 (0.50-2.86)<br>Total Tocopherol (circulating): 0.75 (0.33-1.72)	Age, pack-years of cigarette smoking, serum triglyceride and hours since last meal

Castelao et al. 2004, USA[12]	Matched case-control (PCC)	56.3 (cases) 56.4 (controls)	3184 (1592), both	---	Interview	Vitamin C (diet) 0.73 (0.56-0.95)	Sex, date of birth, race, level of education, number of cigarettes smoked per day, number of years of smoking, smoking status in reference year, lifetime use of nonsteroidal anti-inflammatory drugs, and number of years employed as a hairdresser/barber
Holick et al. 2005, USA[13]	Cohort, Nurses' Health Study	30-55	88796 (237), women	20	FFQ	Vitamin C (total) 1.03 (0.69-1.53) Vitamin E (total) 1.26 (0.85-1.89) Vitamin C (supplement) 1.09 (0.70-1.69) Vitamin E (supplement) 0.72 (0.37-1.42)	Age, pack-years of cigarette smoking, current smoking, and total caloric intake
Hung et al. 2006, USA[14]	Case-control (PCC)	60.2	257 (84), both	---	---	α-Tocopherol (circulating): 0.91 (0.17-4.95) γ-Tocopherol (circulating): 1.81 (0.38-8.64)	Age, sex, pack-years and education
Kellen et al. 2006, Belgium[15]	Case-control (PCC)	67.67 (cases) 64.24 (controls)	540 (178), both	---	Interview	Vitamin C (diet) 0.65 (0.39-1.11) Vitamin E (diet) 0.68 (0.41-1.13)	Sex, age, smoking status, number of cigarettes smoked per day, number of years smoking and occupational exposure to polycyclic aromatic hydrocarbons or aromatic amines
Liang et al. 2008, USA[16]	Matched case-control (PCC)	63.1 (cases) 62.6 (controls)	675 (386), both	---	Interview	α-Tocopherol (circulating): 0.50 (0.3-0.78) γ-Tocopherol (circulating): 1.52 (0.95-2.44) α-Tocopherol (diet):	Age, sex, ethnicity, smoking status, number of cigarettes smoked per day, smoking duration, and plasma cholesterol (for circulating)

						0.85 (0.54-1.32)<br>γ-Tocopherol (diet):<br>1.18 (0.71-1.97)	
Roswall et al. 2009, Denmark[17]	Cohort, Danish Diet, Cancer and Health Study	50-64	55557 (322), both	10.6	FFQ	Vitamin C (total)<br>1.23 (0.87-1.75)<br>Vitamin C (diet)<br>0.99 (0.70-1.39)<br>Vitamin C (supplement)<br>1.19 (0.86-1.66)<br>Vitamin E (total)<br>1.12 (0.78-1.59)<br>Vitamin E (diet)<br>1.07 (0.76-1.51)<br>Vitamin E (supplement):<br>0.97 (0.66-1.44)	Total intake of the three other micronutrients (vitamin C, vitamin E, folate and beta-carotene) as well as dietary intake for the supplemental intake and supplemental intake for the dietary intake and further for smoking status, smoking duration, smoking intensity, possible cessation and when, passive smoking, and work exposure
Brinkman et al. 2010, USA[18]	Case-control (PCC)	62 (cases) 60.7 (controls)	661 (322), both	---	Interview	Vitamin D (total)<br>0.58 (0.31-1.06)<br>Vitamin E (total)<br>0.66 (0.36-1.20)<br>Vitamin C (total)<br>0.78 (0.45-1.38)	Age, sex, smoking status, pack years smoked, and total energy intake
Brinkman et al. 2011, Belgium[19]	Case-control (PCC)	67.6 (cases) 64.2 (controls)	586 (200), both	---	FFQ	Vitamin D (total)<br>1.03 (0.62-1.72)	Age, sex, smoking status, number of cigarettes smoked per day, number of years of cigarette smoking and exposure to occupational carcinogens and energy intake
Amaral et al. 2012, Denmark[20]	Matched case-control (HCC)	68 (cases) 66 (controls)	2153 (1125), both	---	---	Vitamin D (circulating)<br>0.55 (0.35-0.84)	Age, sex, ethnic origin, region, smoking status, and season of blood collection

Mondul et al. 2012, Finland[21]	Cohort (NCC), ATBC cohort	58.6	500 (250), men smokers	11	---	Vitamin D (circulating) 0.61 (0.36-1.05)	Age, date of baseline blood draw, number of cigarettes per day, years of smoking, and vitamin D binding protein
Mondul et al. 2012, USA[22]	Cohort (NCC), PLCO Cancer Screening Study	64	733 (369), both	13	---	Vitamin D (circulating) 1.19 (0.74-1.92)	Age, race, sex, and date of blood collection, smoking status, pack-years of smoking, dairy consumption, and use of aspirin or ibuprofen
Ros et al. 2012, Europe[23]	Cohort (NCC), EPIC	58.5	1712 (856), both	11.0	FFQ	Vitamin C (circulating) 0.85 (0.61-1.17)<br>Vitamin C (diet) 1.11 (0.82-1.51)	Sex, age at baseline, study center, date and time of blood collection, fasting status, smoking status, duration, and intensity
Afzal et al. 2013, Denmark[24]	Cohort, Copenhagen City Heart Study	20-100	9791 (112), both	28	---	Vitamin D (circulating) 0.78 (0.65-0.94)	Age, sex, pack-years, BMI, alcohol consumption, leisure time and work-related physical activity, and duration of education
Park et al. 2013, USA[25]	Cohort, Multiethnic Cohort Study	Men: 60.2<br>Women: 59.7	Men: 83694 (429)<br>Women: 102191 (152)	12.5	FFQ	Vitamin C (diet)<br>Men: 0.83 (0.62-1.11)<br>Women: 0.46 (0.28-0.73)<br>Vitamin E (diet)<br>Men: 0.97 (0.74-1.27)<br>Women: (0.48 (0.29-0.78)	Age at cohort entry, ethnicity, total energy intake, first-degree family history of bladder cancer, employment in a high-risk industry, smoking status, average number of cigarettes, squared average number of cigarettes, number of years smoked (time dependent), number of years since quitting (time dependent), interactions of ethnicity with smoking status, average number of cigarettes, and squared average number of cigarettes

ATBC: Alpha-Tocopherol, Beta-Carotene Cancer Prevention (ATBC) Study, BMI: body mass index, EPIC: European Prospective Investigation into Cancer and Nutrition, FFQ: food frequency questionnaire, HCC: hospital-based case-control study, HPFS: Health Professionals Follow-Up Study, NCC: nested case-control study, PCC: population-based case-control study, PLCO: Prostate, Lung, Colorectal, and Ovarian Cancer Screening Trial

Supplementary table 2 Pooled results on vitamin C and risk of bladder cancer

	Diet plus supplement				Diet				Supplement			
	N	RR (95% CI)	I2 (%)	P[a]	N	RR (95% CI)	I2 (%)	P[a]	N	RR (95% CI)	I2 (%)	P[a]
Total	8	0.86 (0.67-1.10)	52.5		14	**0.84 (0.73-0.98)**	47.5		9	0.87 (0.69-1.11)	64.9	
By design				0.18				0.28				0.28
Cohort	3	1.05 (0.85-1.31)	0.00		7	0.91 (0.73-1.13)	61.5		6	0.97 (0.77-1.22)	51.9	
Case-control	5	0.71 (0.48-1.05)	56.0		7	**0.77 (0.65-0.92)**	7.50		3	0.64 (0.31-1.34)	80.5	
By age (years)				0.76				0.96				0.67
≥60	4	0.82 (0.56-1.19)	41.1		8	**0.84 (0.73-0.98)**	0.00		4	0.81 (0.55-1.20)	65.1	
<60	4	0.89 (0.62-1.27)	67.7		6	0.83 (0.61-1.12)	76.4		5	0.92 (0.66-1.28)	69.5	
Country				0.66				0.15				0.34
USA	6	0.79 (0.58-1.08)	54.8		7	**0.72 (0.59-0.88)**	27.3		7	0.80 (0.58-1.09)	70.4	
Europe	1	1.23 (0.87-1.74)	---		6	0.99 (0.83-1.18)	38.0		2	1.11 (0.87-1.42)	0.00	
Japan	1	0.81 (0.49-1.35)	---		1	0.74 (0.43-1.26)	000		-	---	---	
Assessment method				0.18				0.67				0.28
Interview	4	0.71 (0.48-1.05)	56.0		6	**0.80 (0.68-0.95)**	0.00		3	0.64 (0.31-1.34)	80.5	
FFQ	3	1.05 (0.85-1.31)	0.00		8	0.86 (0.69-1.07)	64.5		6	0.97 (0.77-1.22)	51.9	
Number of cases				0.44				0.28				0.22
≥300	3	1.00 (0.77-1.30)	63.2		8	0.89 (0.74-1.07)	62.3		4	1.05 (0.84-1.32)	35.8	
<300	5	0.76 (0.52-1.13)	16.8		6	**0.74 (0.58-0.93)**	0.00		5	0.72 (0.47-1.10	71.4	
Number of covariates[b]				0.42				**0.01**				0.89
≥4	5	0.80 (0.62-1.03)	33.4		6	**0.69 (0.58-0.82)**	13.7		4	0.84 (0.55-1.29)	74.4	
<4	3	0.96 (0.57-1.61)	66.2		8	1.01 (0.88-1.15)	0.00		5	0.89 (0.65-1.23)	63.9	

a: P values were obtained from meta-regression

b: Number of covariates adjusted including 7 covariates: age, sex, smoking, dietary factors, occupational factors and others (education and physical activity)

Supplementary table 3 Pooled results on vitamin E and risk of bladder cancer

	Diet plus supplement				Diet				Supplement			
	N	RR (95% CI)	I2 (%)	P[a]	N	RR (95% CI)	I2 (%)	P[a]	N	RR (95% CI)	I2 (%)	P[a]
Total	6	0.82 (0.62-1.08)	55.2		9	**0.80 (0.68-0.94)**	33.0		7	**0.78 (0.64-0.94)**	0.60	
By design				0.22				0.14				0.18
Cohort	3	0.96 (0.64-1.45)	73.1		5	0.86 (0.69-1.08)	51.3		6	0.82 (0.67-1.01)	0.00	
Case-control	3	**0.65 (0.47-0.90)**	0.00		4	**0.67 (0.52-0.86)**	0.00		1	**0.51 (0.29-0.89)**	---	
By age (years)				0.43				0.65				0.48
≥60	2	**0.67 (0.46-0.99)**	68.1		5	0.85 (0.72-1.01)	0.00			0.87 (0.62-1.24)	0.00	
<60	4	0.88 (0.61-1.27)	0.00		4	0.74 (0.52-1.04)	60.9			**0.73 (0.57-0.93)**	9.70	
Country				0.96				0.84				0.10
USA	4	0.77 (0.53-1.13)	61.0		3	0.68 (0.42-1.11)	71.0			**0.66 (0.52-0.85)**	0.00	
Europe	1	0.68 (0.41-1.12)	---		5	0.86 (0.73-1.02)	0.00			1.00 (0.73-1.36)	0.00	
Japan	1	1.12 (0.78-1.59)	---		1	0.65 (0.39-1.09)	---		--	---	---	
Assessment method				0.22				0.29				0.18
Interview	3	**0.65 (0.47-0.90)**	0.00		3	**0.69 (0.53-0.90)**	0.00		1	**0.51 (0.29-0.89)**	--	
FFQ	3	0.96 (0.64-1.45)	73.1		6	0.84 (0.67-1.04)	48.2		6	0.82 (0.67-1.01)	0.00	
Number of cases				0.88				**0.03**				0.24
≥300	3	0.80 (0.54-1.19)	62.2		5	0.92 (0.79-1.06)	0.00		4	0.85 (0.67-1.09)	5.30	
<300	3	0.83 (0.52-1.34)	64.6		4	**0.59 (0.46-0.77)**	0.00		3	**0.65 (0.47-0.90)**	0.00	
Number of covariates[b]				0.31				0.25				0.13
≥4	5	0.76 (0.56-1.02)	49.8		5	**0.69 (0.52-0.92)**	47.1			**0.64 (0.48-0.85)**	0.00	
<4	1	1.12 (0.78-1.59)	---		4	0.89 (0.74-1.07)	0.00			0.92 (0.71-1.19)	0.00	

a: P values were obtained from meta-regression

b: Number of covariates adjusted including 7 covariates: age, sex, smoking, dietary factors, occupational factors and others (education and physical activity)

Supplementary table 4 Subgroup analysis by smoking status and sex[a]

Subgroup	Vitamin C			Vitamin D			Vitamin E		
	N[b]	RR (95% CI)	I2 (%)	N[b]	RR (95% CI)	I2 (%)	N[b]	RR (95% CI)	I2 (%)
Among smokers	10	0.92 (0.80-1.05)	67.7	4	**0.72 (0.55-0.93)**	0.00	9	**0.77 (0.64-0.92)**	68.8
Among non-smokers	5	0.94 (0.73-1.20)	36.4	1	1.04 (0.50-2.16)	---	4	1.00 (0.94-1.05)	0.00
Among men	9	0.95 (0.79-1.14)	43.1	2	0.84 (0.49-1.43)	---	9	**0.75 (0.65-0.86)**	0.00
Among women	4	0.74 (0.43-1.27)	75.1	---	---	---	4	0.74 (0.42-1.32)	69.2

a: We combined the result from diet and/or supplement and circulating levels (α-Tocopherol for vitamin E) to increase the number of studies in each subgroup analysis, and the summary estimates were presented in highest vs. lowest levels.
b: number of studies

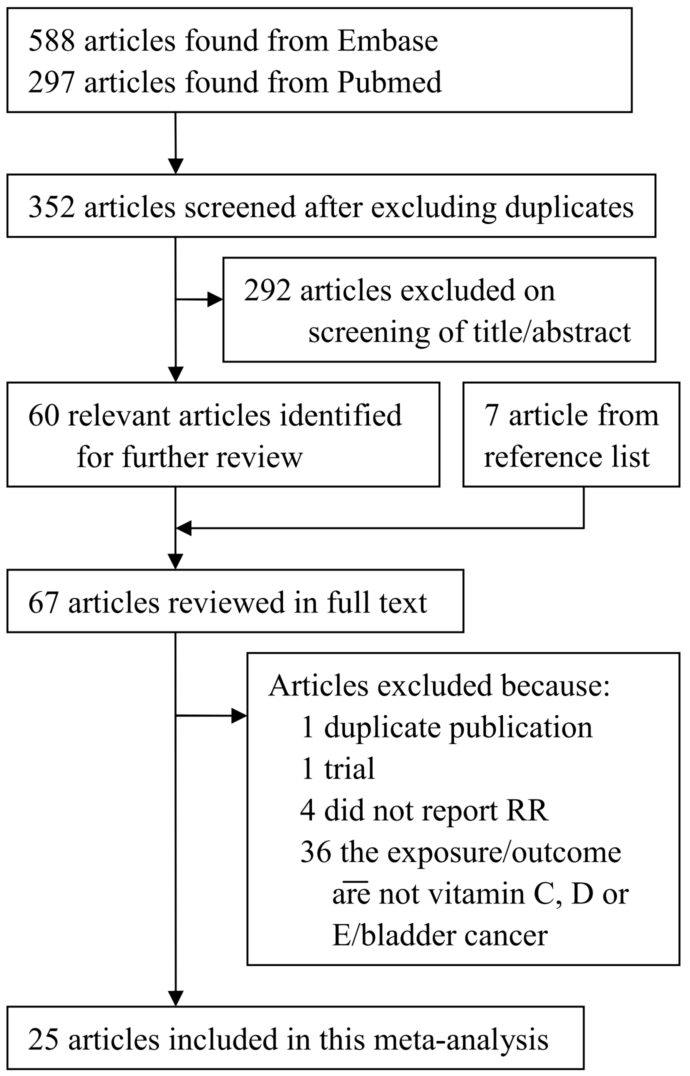

Supplementary figure 1 Flow chart for study inclusion

Supplementary references

1. Helzlsouer, K. J., Comstock, G. W. & Morris, J. S. Selenium, lycopene, alpha-tocopherol, beta-carotene, retinol, and subsequent bladder cancer. *Cancer Res.* **49**, 6144-6148 (1989).
2. Nomura, A. M., Kolonel, L. N., Hankin, J. H. & Yoshizawa, C. N. Dietary factors in cancer of the lower urinary tract. *Int J Cancer.* **48**, 199-205 (1991).
3. Riboli, E. *et al.* Diet and bladder cancer in Spain: a multi-centre case-control study. *Int J Cancer.* **49**, 214-219 (1991).
4. Shibata, A., Paganini-Hill, A., Ross, R. K. & Henderson, B. E. Intake of vegetables, fruits, beta-carotene, vitamin C and vitamin supplements and cancer incidence among the elderly: a prospective study. *Br J Cancer.* **66**, 673-679 (1992).
5. Bruemmer, B., White, E., Vaughan, T. L. & Cheney, C. L. Nutrient intake in relation to bladder cancer among middle-aged men and women. *Am J Epidemiol.* **144**, 485-495 (1996).
6. Michaud, D. S. *et al.* Prospective study of dietary supplements, macronutrients, micronutrients, and risk of bladder cancer in US men. *Am J Epidemiol.* **152**, 1145-1153 (2000).
7. Wakai, K. *et al.* Foods and nutrients in relation to bladder cancer risk: a case-control study in Aichi Prefecture, Central Japan. *Nutr Cancer.* **38**, 13-22 (2000).
8. Zeegers, M. P., Goldbohm, R. A. & van den Brandt, P. A. Are retinol, vitamin C, vitamin E, folate and carotenoids intake associated with bladder cancer risk? Results from the Netherlands Cohort Study. *Br J Cancer.* **85**, 977-983 (2001).
9. Jacobs, E. J. *et al.* Vitamin C and vitamin E supplement use and bladder cancer mortality in a large cohort of US men and women. *Am J Epidemiol.* **156**, 1002-1010 (2002).
10. Michaud, D. S. *et al.* Intakes of fruits and vegetables, carotenoids and vitamins A, E, C in relation to the risk of bladder cancer in the ATBC cohort study. *Br J Cancer.* **87**, 960-965 (2002).
11. Nomura, A. M., Lee, J., Stemmermann, G. N. & Franke, A. A. Serum vitamins and the subsequent risk of bladder cancer. *J Urol.* **170**, 1146-1150 (2003).
12. Castelao, J. E. *et al.* Carotenoids/vitamin C and smoking-related bladder cancer. *Int J Cancer.* **110**, 417-423 (2004).
13. Holick, C. N. *et al.* Intake of fruits and vegetables, carotenoids, folate, and vitamins A, C, E and risk of bladder cancer among women (United States). *Cancer Causes Control.* **16**, 1135-1145 (2005).
14. Hung, R. J. *et al.* Protective effects of plasma carotenoids on the risk of bladder cancer. *J Urol.* **176**, 1192-1197 (2006).
15. Kellen, E., Zeegers, M. & Buntinx, F. Selenium is inversely associated with bladder cancer risk: a report from the Belgian case-control study on bladder cancer. *Int J Urol.* **13**, 1180-1184 (2006).
16. Liang, D. *et al.* Plasma vitamins E and A and risk of bladder cancer: a case-control analysis. *Cancer Causes Control.* **19**, 981-992 (2008).
17. Roswall, N. *et al.* Micronutrient intake and risk of urothelial carcinoma in a prospective Danish cohort. *Eur Urol.* **56**, 764-770 (2009).

18. Brinkman, M. T. *et al.* Minerals and vitamins and the risk of bladder cancer: results from the New Hampshire Study. *Cancer Causes Control.* **21**, 609-619 (2010).
19. Brinkman, M. T. *et al.* Dietary intake of micronutrients and the risk of developing bladder cancer: results from the Belgian case-control study on bladder cancer risk. *Cancer Causes Control.* **22**, 469-478 (2011).
20. Amaral, A. F. *et al.* Plasma 25-hydroxyvitamin D(3) and bladder cancer risk according to tumor stage and FGFR3 status: a mechanism-based epidemiological study. *J Natl Cancer Inst.* **104**, 1897-1904 (2012).
21. Mondul, A. M., Weinstein, S. J., Virtamo, J. & Albanes, D. Influence of vitamin D binding protein on the association between circulating vitamin D and risk of bladder cancer. *Br J Cancer.* **107**, 1589-1594 (2012).
22. Mondul, A. M., Weinstein, S. J., Horst, R. L., Purdue, M. & Albanes, D. Serum vitamin D and risk of bladder cancer in the Prostate, Lung, Colorectal, and Ovarian (PLCO) Cancer Screening trial. *Cancer Epidemiol Biomarkers Prev.* **21**, 1222-1225 (2012).
23. Ros, M. M. *et al.* Plasma carotenoids and vitamin C concentrations and risk of urothelial cell carcinoma in the European Prospective Investigation into Cancer and Nutrition. *Am J Clin Nutr.* **96**, 902-910 (2012).
24. Afzal, S., Bojesen, S. E. & Nordestgaard, B. G. Low plasma 25-hydroxyvitamin D and risk of tobacco-related cancer. *Clin Chem.* **59**, 771-780 (2013).
25. Park, S. Y. *et al.* Fruit and vegetable intakes are associated with lower risk of bladder cancer among women in the Multiethnic Cohort Study. *J Nutr.* **143**, 1283-1292 (2013).
